# Illicit drug use in Australia during the COVID-19 pandemic

**DOI:** 10.7189/jogh.12.03026

**Published:** 2022-05-30

**Authors:** Natasha M Loi, Einar B Thorsteinsson, Kylie Rice, Adam J Rock

**Affiliations:** School of Psychology, University of New England, Armidale NSW, Australia

The use of illicit drugs is associated with numerous negative psychological, physiological, financial, social, and legal effects [1]. The restrictions associated with COVID-19 have exacerbated these outcomes for many people around the world, with an increase in anxiety and depression and reduced overall well-being being evident [2].

Restrictions associated with the COVID-19 pandemic have limited travel and people’s day-to-day movement, resulting in an inability to interact in person with friends and family, leading to a marked reduction in overall well-being for many people. The changes brought about by these restrictions to what were often previously regarded as prosaic activities have had several flow-on effects, including alterations in how people work, socialise, and engage in any number of licit and illicit pursuits. The restrictions imposed on mobility have particularly affected how people use illicit drugs.

## FACTORS CONTRIBUTING TO CHANGES IN DRUG USE

Studies have suggested that the societal changes resulting from the pandemic have altered the use of various drugs. Langfield et al. [3] suggest three potential reasons why a possible increase in community drug use may be seen during the pandemic. First, the closure of many industries may lead to an increase in unemployment levels, particularly among young Australians [4], which, accompanied by social distancing restrictions and the closure of entertainment venues, could potentially increase recreational drug use. Second, due to the precarity of many people’s employment and economic circumstances, as well as the unprecedented nature of the pandemic itself, some people may turn to drug use as a coping strategy. Third, a reduction in treatment availability due to restrictions affecting and, in some cases, even halting some services may lead to an increase in drug use. The fact that people may be less likely to want to enter a medical facility during a pandemic or that their usual treatment service provider may have closed due to restrictions places additional challenges on remaining engaged with treatment. Linas et al. [5] suggest that an increase in help-seeking during this time is unlikely, even if other options such as telehealth were available.

Thus, the social distancing policies implemented by most of the world’s governments in order to mitigate the spread of COVID-19 have created a unique environment in which people are isolated from one another, resulting in an increase in many mental health problems, but also a general increase in substance use [6,7].

**Figure:**
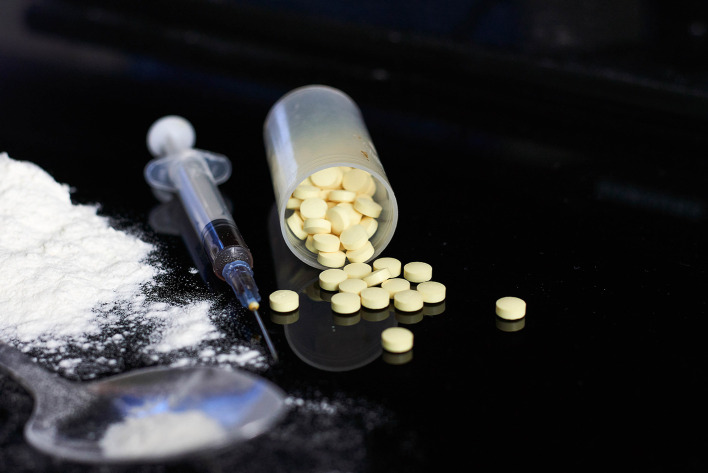
Photo: Drug addiction concept – pills, syringe and heroin. Source: from Marco Verch, https://www.flickr.com/photos/30478819@N08/50717084261/, CC BY 2.0.

## DRUG USE BEFORE AND DURING THE PANDEMIC

Research conducted in 2020 examining drug usage during the pandemic found that while almost 26% of participants indicated that their use had decreased, almost 18% said their consumption had increased [8], suggesting that there may have been variable changes in drug use during the pandemic.

In 2019, the most used drugs in Australia were cannabis and methamphetamines, used more frequently (37% and 17% using these at least once a week or more, respectively) than other drugs such as cocaine (6.7%) or ecstasy (4.5%) [9]. However, several studies have shown conflicting findings with respect to the use of exactly which drugs have increased, decreased, or stayed the same. For example, during the pandemic, the use of drugs in Australia usually used in social situations such as MDMA and cocaine either decreased or stayed the same [10]. Conversely, according to statistics obtained from the National Wastewater Drug Monitoring Program [11], increases in the use of cocaine and MDMA were found between April and August 2020, suggesting a lack of consensus between objective measures of drug use and survey data. A global study conducted by Farhoudian et al. [12] found that participants in Australia indicated an increase in the use of cannabis, amphetamines, and sedatives during the pandemic. Fry et al. [13], though, found that methamphetamine use appeared to have decreased in 2020 compared to the same period in 2019. This lack of consensus highlights the heterogeneous effect that the pandemic has had on the consumption of illicit drugs both across individuals as well as substances. For instance, the use of more social drugs (eg, MDMA) may have decreased because of the restrictions around gatherings during the lockdowns. Conversely, the use of non-social drugs (eg, amphetamines, sedatives) may have increased given the fact that people were isolated from one another.

Given that COVID-19 may not be fully eradicated through vaccines (eg, due to different variants of COVID-19), it will be important for policy makers to carefully consider its long-lasting impact on drug use and procurement.

## PROCUREMENT OF DRUGS

There have been suggestions of a significant shift in the procurement of illicit drugs during the COVID-19 pandemic, as increased lockdowns and other restrictions relating to the movement of people and disruptions to freight have altered how people purchase drugs [14,15]. While most people have continued to purchase drugs face-to-face, many have reduced this method of procurement, purchase drugs less often, and have bought drugs in larger quantities in response to pandemic restrictions [15]. Alternatively, the absence of a person’s regular supply of drugs, potentially resulting in the experience of withdrawal, may lead to desperate measures – including engaging in riskier than usual behaviours in order to procure drugs. Such behaviours may result in an increased likelihood of arrest or other legal outcomes [14].

## CLINICAL IMPLICATIONS

In addition to possible increases in risky behaviour to procure drugs during COVID-19 restrictions, there are clinical implications in changes to drug use. First, the extensive mental health impacts of the pandemic, combined with the considerable disruptions to lifestyle and social supports, have reduced well-being [16]. In response, substance users may apply less effective coping strategies [17] and may turn to illicit drugs to manage mental health and compounding life difficulties [18]. Substance users in isolation may increase the dosage and frequency, enabled by the larger quantities being purchased at one time during the pandemic [15], and may increase the chance of overdose, tolerance, and dependence [19]. Furthermore, the reduced availability of illicit drugs may also result in procurement from less reliable sources, possibly reducing the quality and safety of available drugs, and increasing the chance of contamination, substitution, adulteration and consumption of more harmful substances [18]. Compounding this increased risk is a disruption in the availability of harm reduction facilities (eg, syringe service programs) [19], and decreased support services and treatment options due to COVID-19 [18], reducing the chance of effective risk management. Furthermore, people who use substances often have comorbid physical and mental health issues, additional vulnerabilities (eg, employment or housing issues), and are at a greater risk of contracting COVID-19 and having serious health effects from the virus [20,21]. These factors may increase stress and anxiety in this population, and perpetuate the maladaptive cycle of substance use as a coping strategy to reduce distress.

## CONCLUSION

Societal changes due to the COVID-19 pandemic, including changes in employment status, available coping strategies (eg, social support from friends and family may be more limited), and availability of drug treatment options have appeared to affect the use of illicit drugs in Australia.

The procurement method of choice still appears to be predominantly face-to-face but occurring less frequently, leading to larger quantities of drugs being bought each time which is in line with pandemic restrictions attempting to reduce social contact. Many of the effects of COVID-19 on the drug market, however, may be temporary, and given the lack of government policies or red tape to contend with, the drug market could likely adapt. As a result, factors other than the pandemic may have more influence on changes in demand, drugs of choice, and procurement, including economic factors such as government budgets allocated to drug enforcement. In conclusion, how people procure and use illicit substances once the pandemic ends and life returns to relative normality still remains to be seen. Like many behaviours that have acclimated to the “new normal”, drug-related behaviours will presumably return to something resembling pre-2020 life. The last two years have clearly shown, though, that where there is drug demand there will be a drug supply, pandemic or not. If societies want to reduce drug-related harm, then changes to legal frameworks based on evidence-based harm reduction health research are needed, with politics removed from the equation.
